# Evaluation and Management of Complications of Endovascular Aneurysm Repair of the Thoracic Aorta

**DOI:** 10.7759/cureus.36930

**Published:** 2023-03-30

**Authors:** Stephen J Bordes, Baris Vefali, Lisandro Montorfano, Philip Bongiorno, Mark Grove

**Affiliations:** 1 Surgery, Louisiana State University Health Sciences Center, New Orleans, USA; 2 Cardiology, St. Michael Medical Center, Newark, USA; 3 Surgery, Vanderbilt University Medical Center, Nashville, USA; 4 Surgery, Cleveland Clinic Florida, Weston, USA; 5 Cardiothoracic Surgery, Cleveland Clinic Florida, Weston, USA; 6 Vascular Surgery, Cleveland Clinic Florida, Weston, USA

**Keywords:** tevar complications, endovascular surgery, vascular surgery, endovascular aneurysm repair, tevar

## Abstract

Thoracic endovascular aortic repair (TEVAR) has become the standard of care for descending thoracic aortic pathology as the procedure has a historically low rate of reintervention and a high rate of success. However, TEVAR can be associated with complications such as endoleak, upper extremity limb ischemia, cerebrovascular ischemia, spinal cord ischemia, and post-implantation syndrome.

An 80-year-old man with a history of complex thoracic aortic aneurysms underwent repair of a large thoracic aneurysm with a frozen elephant trunk procedure in 2019 at an outside institution. The proximal aortic graft extended to the arch and the innominate and left carotid artery were implanted into the distal portion of the graft. The endograft, extending from the proximal graft to the descending thoracic aorta, was fenestrated to maintain left subclavian artery flow. In an attempt to gain a seal at the fenestration, a Viabahn graft (Gore, Flagstaff, AZ, USA) was inserted. A type III endoleak was identified postoperatively at the fenestration, and a second Viabahn graft was required to gain a seal during the initial hospitalization. In 2020, an endoleak persisted at the fenestration on follow-up imaging, but the aneurysmal sac was stable. No intervention was recommended. The patient later presented to our institution with three days of chest pain. A type III endoleak at the level of the subclavian fenestration persisted with significant enlargement of the aneurysm sac. The patient underwent an urgent repair of the endoleak. This consisted of covering the fenestration with an endograft and left carotid to subclavian bypass. Subsequently, the patient developed a transient ischemic attack (TIA) due to kinking and extrinsic compression by the large aneurysm sac of the proximal left common carotid artery, requiring a right carotid to left carotid-axillary graft bypass.

This report with a literature review discusses TEVAR complications and outlines methods to approach them. TEVAR complications and their management should be firmly understood to improve overall treatment outcomes.

## Introduction

Thoracic endovascular aortic repair (TEVAR) is the gold standard when treating descending thoracic aortic aneurysms [1]. The procedures are widely used and generally efficacious; however, they do carry risks such as endoleak, upper extremity limb ischemia, cerebrovascular ischemia, and spinal cord ischemia [2]. We describe a complex case of an 80-year-old man who required an urgent endovascular repair due to newly developed symptoms of pain from an enlarging aneurysm sac caused by a chronic type III endoleak. The patient’s management was complicated by the need to maintain antegrade flow to the left subclavian artery and the subsequent recognition of inadequate inflow to the left common carotid artery. Furthermore, we review TEVAR complications and the recommended treatment approaches for each of them.

## Case presentation

**Figure 1:**
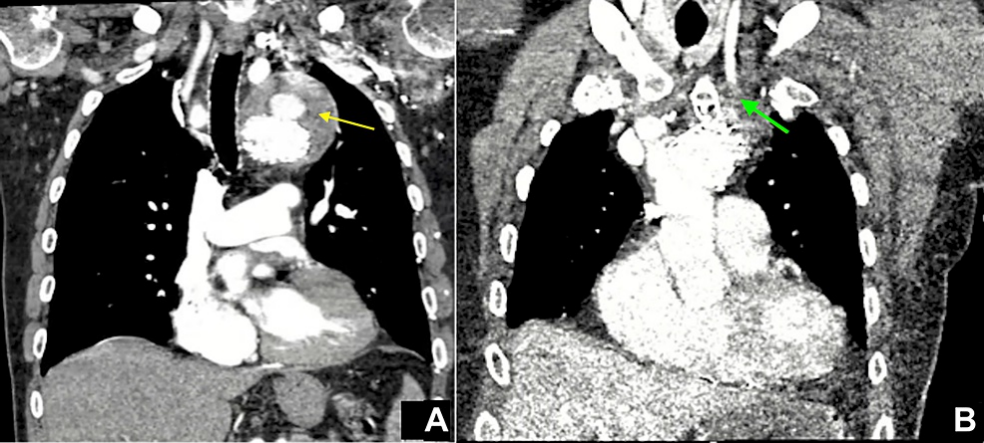
(A) Type III endoleak in the distal aortic arch resulting in increased aneurysmal dilatation of the excluded portion of the aneurysmal sac in the proximal descending thoracic aorta (yellow arrow). (B) Severe focal stenosis of the proximal left common carotid artery from extrinsic compression between the aortic aneurysm sac and sternum/clavicular head (green arrow).

An 80-year-old man came to the hospital following three days of chest pain. He underwent repair of a large thoracic aneurysm with a frozen elephant trunk procedure in 2019 at an outside institution. The polytetrafluoroethylene (PTFE) endograft extended from just beyond the re-implanted left common carotid artery. The endograft was fenestrated at the left subclavian artery ostium, with the insertion of a Viabahn graft (Gore, Flagstaff, AZ, USA) to gain a seal. During the index hospitalization, an endoleak was identified at the fenestration, and a second Viabahn graft was placed to attempt to seal. The patient had a follow-up computed tomography (CT) scan in August 2020. At that time, an endoleak was noticed, but the aneurysmal sac was stable. As such, no intervention was recommended. On presentation to our institution with chest pain, computed tomography with angiography (CTA) showed a type III endoleak in the distal aortic arch and an increased aneurysmal dilatation (9.5 × 8.6 cm) of the excluded portion of the aneurysmal sac in the proximal descending thoracic aorta (Figure 1a).

Our proposed strategy was to cover the left subclavian artery fenestrated endograft with a new endograft, plugging the left subclavian artery proximally with an Amplatzer device (Abbott, Chicago, IL, USA) and creating a left carotid-to-subclavian bypass to maintain antegrade perfusion of the left subclavian artery. The left femoral artery was exposed through a transverse incision. A pigtail catheter was advanced over a Glidewire into the ascending aorta. The initial arch aortogram demonstrated excellent perfusion of the innominate artery and filling of the left carotid artery. Proximally, the left carotid artery was posteriorly oriented, and while the brisk filling was noted on the angiogram, the proximal portion was not visualized. An 18-mm Amplatzer plug was advanced into position to maintain perfusion of the left vertebral artery and remain outside of the existing endograft proximally. The plug was deployed without difficulty and in the proper position. Preparations for new endograft placement were then undertaken. A compliant balloon was brought into the arch. The balloon was inflated just proximally to the left subclavian artery stent, the presumed site of the endoleak. The Viabahn stent was directed posteriorly with the balloon. The Viabahn appeared to be quite mobile at that position. A 40 × 40 × 95 mm Medtronic Valiant Navion (Medtronic, Minneapolis, MN, USA) graft was deployed and aligned with the previous proximal endograft. This provided approximately 20 mm of coverage proximal to the fenestration. The Viabahn stent was crimped against the original endograft by the new endograft. A repeat aortogram was performed, and no evidence of type I or type III endoleaks was seen. A left carotid-axillary bypass was performed. The axillary artery was accessed through a transverse incision inferior to the clavicle and noted to be non-pulsatile due to the proximal occlusion. The carotid artery was exposed using a standard anterior sternocleidomastoid incision. The carotid bifurcation was relatively low, and the proximal extension of the incision nearly extended to the level of the sternal notch to afford exposure of the proximal common carotid artery. A tunnel was made to accommodate the 8-mm ring-enforced polytetrafluoroethylene (PTFE) graft deep to the clavicle. Distal and proximal anastomoses were performed end-to-side using continuous 5-0 Prolene sutures (Ethicon, New Brunswick, NJ, USA). The patient’s brachial pulse was palpable at the conclusion of the surgery.

**Figure 2:**
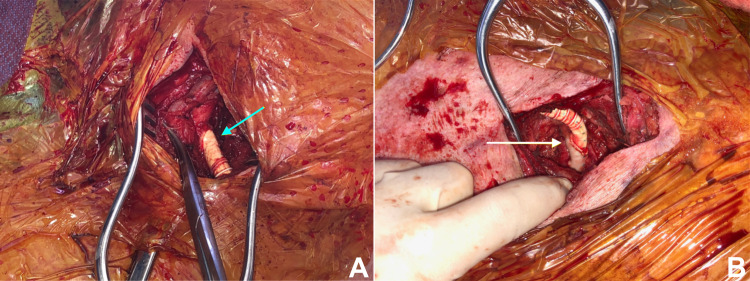
(A) Right carotid to graft anastomosis (blue arrow). (B) Graft-to-graft anastomosis (white arrow).

On postoperative day (POD) one, the patient developed an altered mental status and became unresponsive with right-sided neglect and hemiparesis. A CTA was performed and confirmed that the carotid-axillary bypass graft was widely patent. However, there was a loss of visible luminal contrast involving the proximal left common carotid artery due to compression as it coursed between the aneurysmal sac and sternum/left clavicular head (Figure 1b). Endovascular options were discussed and deemed impractical as the stent would likely be crushed due to compression. The patient was therefore taken back to the operating room. The right common carotid artery was exposed through a standard anterior sternocleidomastoid incision. The decision was made to perform the distal anastomosis to the carotid-axillary graft to restore antegrade perfusion of both the left upper extremity and the common carotid artery (Figure 2). The previous pectoral incision was reopened, and the graft was readily exposed. A tunnel was created anterior to the trachea to connect the two incisions in the subcutaneous/subplatysmal plane. A 7 mm ring-reinforced PTFE graft was chosen for the bypass. This graft was positioned in the aforementioned tunnel. An end-to-side anastomosis to the right common carotid artery was performed using a continuous 6-0 PTFE suture. The attention was then directed to the left side. End-to-side graft-to-graft anastomosis was performed again using a continuous 6-0 PTFE suture. At the end of the case, the patient had a palpable left radial pulse. The remainder of the hospital stay was uneventful, and the patient had no residual neurological deficits.

## Discussion

The complication rate of TEVAR has been estimated to be as high as 38%, and the most common complications include endoleak, upper extremity limb ischemia, cerebrovascular ischemia, spinal cord ischemia, and post-implantation syndrome [2]. Endoleaks are the most common causes of TEVAR reintervention and are defined as functional failures in the deployed endografts, allowing for the persistent flow of blood into the excluded aneurysm sac. There are five types of endoleaks, which are classified according to their specific functional failure (Table 1). Type I is caused by inadequate fixation of the proximal (Ia) or distal (Ib) attachment sites of the deployed endograft [3]. This can be due to many factors, including preoperative mural thrombus, vessel calcification, branching vessels, graft migration, or incorrect sizing of stent-graft materials. Type I endoleaks have been described as occurring both immediately postoperatively and in the late recovery period, dependent on contributing factors. Type II endoleaks are caused by blood entering the aneurysmal sac in a retrograde fashion via patent branching vessels [3]. Often excluded by the endograft, segmental arteries can have retrograde flow via internal thoracic and intercostal arteries, contributing to endoleak and aneurysmal growth. Type III endoleaks are described as junctional interruptions in endograft components (IIIa) or stent-graft material failures, such as fabric tears, suture breakage, or component fracture (IIIb) [3,4]. Type III can also be observed as both early and late onset depending on contributing factors. Type IV and type V endoleaks are extremely rare after TEVAR. Type IV is caused by fabric porosity, which allows for the outflow of plasma into the excluded aneurysm [3]. Type V is due to endotension or aneurysmal expansion without radiological evidence of another source [3]. In a review of 27 studies evaluating TEVAR for aortic dissection, reintervention was required in 15% of cases, with 33% of those due to an endoleak [5]. In all indications for TEVAR, endoleaks have an estimated incidence of 3.9-15%. Data are conflicting as to which type of endoleak is most common. Type I and type II endoleaks are considered the most prevalent by several studies. While Type III endoleak incidence varies, Type IIIa endoleaks occur in over 2% of cases across all modalities [2,5-7].

**Table 1:** Endoleak type and source.

Endoleak type and source	
I	Inadequate graft seal (Ia: proximal graft; Ib: distal graft)
II	Branch to branch, back filling (IIa: inflow to sac only; IIb: inflow and outflow from sac)
III	Graft failure (IIIa: junctional/component separation; IIIb: graft fracture/defect)
VI	Porous graft material
V	Endotension

Once a decision is made to intervene, management of endoleaks varies by type. Type I endoleaks are best handled by extending the proximal and distal portions of the stent graft to include non-diseased portions of the aorta and by using endoanchors, which securely fasten edges [8]. However, the extension of the proximal or distal edges of grafted stents requires consideration of the risk associated with coverage of the left subclavian artery, left carotid artery, or spinal segmental arteries. Type II endoleak treatments include embolization or ligation of the branching artery supplying the retrograde flow to the persistent excluded pathology. Type III endoleaks are managed by the placement of an additional endograft within the lumen. This relines the gap between the previously deployed or defective grafted material and prevents further leakage into the excluded pathology. Types IV and V do not typically require intervention; however, relining the existing graft can prevent the progression of aneurysmal sac expansion [2].

Post-TEVAR left upper extremity limb ischemia may result from complete coverage of the left subclavian artery. Large, extending aortic aneurysms often require a landing zone near the great vessels arising from the aortic arch. Intentionally covering the subclavian origin to ensure a robust proximal seal has been recognized as an acceptable and well-tolerated method to reduce the incidence of type Ia endoleak and strengthen endograft attachment [9]. However, more recent studies continue to debate whether left subclavian coverage has a clinical impact. There is evidence that up to 4% of cases with complete subclavian coverage develop left arm ischemia. Conversely, a retrospective study of all cases of TEVAR in the Nationwide Inpatient Sample (NIS) database revealed data that showed no significant difference in the incidence of upper limb ischemia between TEVAR with and without revascularization of the left subclavian artery [10-12].

Left arm ischemia from subclavian occlusion is typically managed via revascularization with either extra-anatomic bypass or transposition. For extra-anatomic bypass, carotid-subclavian or carotid-axillary bypass is performed using a prosthetic graft. The carotid-axillary bypass has been recommended as an alternative to the carotid-subclavian bypass due to a lower rate of phrenic nerve palsy, a leading concern for that intervention [1]. Subclavian transposition, conversely, has the advantage of obviating the need for a prosthesis, leading to nearly perfect long-term patency, low risk of complication, and even lower permanent morbidity [13,14]. However, subclavian transposition requires extensive dissection for mobilizing the vessels to ensure tension-free anastomosis, limited by both anatomical variants and surgical history [14].

The timing of left subclavian revascularization with or following TEVAR is debated. Revascularization, though recommended for postoperative upper limb ischemia, can potentially increase the morbidity of TEVAR. Left subclavian revascularization can lead to increased stroke, cardiopulmonary complications, and spinal cord ischemia.

Post-TEVAR stroke is thought to be associated with coverage of the carotid and vertebral vessels. Another important cause is embolism, which explains the increased odds related to left subclavian revascularization. Historically, embolic stroke has been observed in 4-8% of TEVAR cases. The risk was regarded as significant but similar when compared to open procedures [2]. However, TEVAR following supra-aortic debranching methods can result in a silent cerebral infarction in up to 21% of cases. The presence of atheromatous disease in the proximal landing zone and its degree of severity were determined to be significant predictors of incidence [15]. Another investigation found that post-TEVAR silent cerebral infarction may be as high as 80% and could be linked to neurocognitive decline [16].

Management of brain hypoperfusion should be managed based on etiology. Standard stroke management protocols should be employed to determine the cause of hypoperfusion post-TEVAR [2]. Symptomatic embolic strokes should be managed per institutional protocols, avoiding tissue plasminogen activator (tPA) due to post-surgical status. Should the etiology be due to left carotid obstruction secondary to elements of aortic grafting, as in this case, carotid-carotid bypass can be therapeutic.

Spinal cord ischemia is a complication of TEVAR due to endograft coverage of segmental artery origins within the descending thoracic aorta [3]. Incidence is estimated to be 2.5-8%, but only transient in over 5% of cases and permanent in approximately 2% of cases. A prior study suggests a direct relationship between the extent of segmental artery coverage and the risk of paraplegia in descending thoracic aortic aneurysm repair with TEVAR [17,18]. Coverage should be limited as much as possible to mitigate risk.

Management of spinal cord ischemia should focus on prevention, as there is no effective treatment for permanent spinal cord damage. Two promising proposed tactics include preoperative spinal drainage and perioperative epidural corticosteroid usage. The use of spinal drainage at the time spinal cord ischemia is diagnosed has been shown to have a weaker effect.

Additionally, there are other factors that should be reviewed to potentially lower the rate of TEVAR reintervention due to common complications. The literature revealed that the initial presentation and complexity of the techniques chosen may directly impact the rate of reintervention.

Initial presentation has been shown to help predict the rate of TEVAR reintervention. A review of 585 patients treated with TEVAR for all indications at one institution found that initial presentation with acute aortic dissection requires reintervention in 21.3% of cases, closely followed by chronic aortic dissection at 16.7%, degenerative aneurysm at 10.8%, and traumatic transection at 8.1% [19]. This differs from a more recent study of 1,037 patients at a single institution that showed chronic aortic dissection to predict reintervention at a rate of 26.5% and acute aortic dissection at 11.2% [20]. It is important to note, however, that type III endoleak following a complex endovascular repair is extremely rare, as is noted in a recent study that examined a group of 4,070 complex endovascular cases, which included cases requiring fenestrated grafts over a 10-year period [21]. It was determined that type III endoleaks did not result in a clinically significant rate of reintervention. Most of the type III endoleaks identified were found at the time of index case admission (4.1%), whereas those found incidentally at a later time occurred <0.1% of the time [21].

Overall, close follow-up for patients after TEVAR is recommended, especially for those with a high-risk initial presentation. Patients should return at the one-month and six-month mark for postoperative non-contrast CT chest imaging to assess operative success and postoperative aneurysmal change. Aneurysmal exclusion should be documented. A review of 585 patients treated with TEVAR for all indications found that 12% required secondary aortic intervention (SAI) at a median of 5.6 months [19]. A different study following all patients undergoing TEVAR over 14 years showed that 14.9% required SAI within a median of five months [20]. These data suggest that problematic pathology may be adequately identified within a six-month follow-up window. Routine maintenance surveillance is suggested at intervals of 6-12 months, which is typically dictated by the institution.

## Conclusions

In this case, we present a complex intervention following TEVAR and a subsequent endoleak in an 80-year-old man, which resulted in excellent clinical outcomes. A type III endoleak was initially managed with the deployment of a second endograft. However, an open approach was ultimately deemed necessary to restore left carotid flow following cerebrovascular complications secondary to aneurysm sac compression. We discuss the literature regarding TEVAR complications and the best practices for treating them. We hope to increase awareness of common TEVAR complications and their management strategies to improve overall clinical outcomes.
